# Financial Self-Efficacy and General Life Satisfaction: The Sequential Mediating Role of High Standards Tendency and Investment Satisfaction

**DOI:** 10.3389/fpsyg.2021.545508

**Published:** 2021-03-17

**Authors:** Jianping Hu, Lei Quan, Yanwei Wu, Jia Zhu, Mingliang Deng, Song Tang, Wei Zhang

**Affiliations:** ^1^Laboratory for Behavioral and Regional Finance, Guangdong University of Finance, Guangzhou, China; ^2^School of Economics and Management, Wuhan University, Wuhan, China; ^3^School of Psychology, South China Normal University, Guangzhou, China; ^4^Center for Studies of Psychological Application, South China Normal University, Guangzhou, China

**Keywords:** financial self-efficacy, general life satisfaction, high standards tendency, investment satisfaction, sequential mediation model

## Abstract

Important strides have been made toward understanding the relationship between self-efficacy and life satisfaction. However, existing studies have largely focused on work and academic domains, leaving self-efficacy in the finance domain less frequently investigated. The present study applied the self-efficacy construct to the finance domain, namely “financial self-efficacy” (FSE), and tested the sequential mediating roles of high standards tendency and investment satisfaction in the relationship between FSE and general life satisfaction. A total of 323 employees from finance-related businesses completed anonymous questionnaires regarding FSE, high standards tendency, investment satisfaction, and general life satisfaction. Results indicated that FSE influenced general life satisfaction through investment satisfaction, and sequentially through high standards tendency and investment satisfaction. These results provide contributions to the current literature on life satisfaction, and positive psychology literature by shedding light on the roles of high standards tendency and investment satisfaction in the relation between FSE and general life satisfaction.

## Introduction

With recent developments in positive psychology, life satisfaction has become a frequently studied topic (Diener, 2000; Peterson et al., 2005; Ginevra et al., 2018; Russo-Netzer et al., 2019). Life satisfaction can be described as a cognitive process, in which individuals assess the quality of their lives based on their own unique standards (Pavot and Diener, 1993). Increasing evidence has revealed that life satisfaction is associated with positive social and emotional functioning (Suldo and Huebner, 2006; Heffner and Antaramian, 2016). Furthermore, as it is an important component in subjective wellbeing, life satisfaction is likely to reflect fulfillment of personal values and goals, acting as a positive indicator of mental health (Steger et al., 2011; Fergusson et al., 2015). Additionally, substantial evidence has identified life satisfaction as a protective factor against a variety of internalizing and externalizing disorders, including depression, anxiety, and substance abuse (Paschali and Tsitsas, 2010; Sun and Shek, 2010; Gigantesco et al., 2019). Thus, given its importance, researchers have devoted considerable efforts to identifying psychological factors that may bolster life satisfaction.

Important strides have been made toward understanding dispositional resources related to life satisfaction. According to the social cognitive model of wellbeing, self-efficacy is one of the central constructs associated with life satisfaction (Lent, 2004). Both general self-efficacy and domain-specific self-efficacy have showed significant and positive associations with life satisfaction (Bandura, 1989, 1997; Lent et al., 2005; Azizli et al., 2015; Garriott et al., 2015; Paciello et al., 2016; Burger and Samuel, 2017). However, although financial behaviors have become one of the most important parts of life, existing studies have largely focused on work and academic domains, leaving self-efficacy in the finance domain less frequently investigated (Gutter and Copur, 2011). Therefore, the current study applied the construct of self-efficacy to the finance domain—financial self-efficacy (FSE) and examined the sequential mediating mechanisms of the association between FSE and life satisfaction.

### The Mediating Role of Investment Satisfaction

Financial self-efficacy (FSE) refers to one’s belief in his or her ability to achieve financial goals (Forbes and Kara, 2010). The social cognitive model of wellbeing proposed a number of mediating paths between self-efficacy (both general and domain-specific) and life satisfaction, including “from personality characteristics – *via* generalized and domain-specific self-efficacy – to domain-specific and life satisfaction” and “from personality characteristics – *via* domain-specific satisfaction – to life satisfaction” (Lent, 2004). These paths imply the mediating effect of domain-specific satisfaction in the association between domain-specific self-efficacy and life satisfaction.

In the finance domain, one potential mechanism underlying the association between FSE and life satisfaction is investment satisfaction. Investment satisfaction describes investors’ subjective evaluations of the quality of their decisions and performance (Asif, 2016). It is similar to, but still different from, “financial satisfaction,” which refers to the overall subjective evaluation of an individual’s financial situation (Ng and Diener, 2014), while investment satisfaction concerns judgments of one’s investment quality. Investment in risky and riskless assets is one of the most important strategies for financial wealth accumulation (Donnelly et al., 2012). Recent evidence has shown that making better investment decisions is significant in enhancing the likelihood of financial independence at a future date (Xiao et al., 2014a). Thus, investment satisfaction is better considered as a separate indicator, not combined with financial satisfaction.

Some indirect evidence has suggested the mediating role of investment satisfaction in the relationship between FSE and general life satisfaction. Core characteristics of FSE may help in the understanding of its relationship with investment satisfaction. Goal orientation inherent in self-efficacy appears to be critical in fostering achievement-relevant behaviors (Bandura, 1997; Brown et al., 2005). Specifically, individuals with a strong sense of FSE may focus their attention and motivation on how to master the investment decision-making process to gain favorable outcomes, thus leading to higher levels of satisfaction with their investment performance (Lee and Mortimer, 2009; Korniotis and Kumar, 2011; Xiao et al., 2014b; Farrell et al., 2016). Moreover, some empirical studies have suggested a positive correlation between FSE and investment satisfaction. For example, perceived financial capability (a variable similar to FSE) was found to have a positive correlation with financial satisfaction (Xiao et al., 2014b). Furthermore, numerous studies have demonstrated positive relationships between domain-specific satisfaction and general life satisfaction. Such positive associations have been identified in many major life domains, including work, academia, and family (Badri et al., 2013; Weber and Huebner, 2015; Cho and Tay, 2016; Sheu et al., 2016). The increasingly important role of financial satisfaction, especially investment satisfaction, in the improvement of modern life calls for more comprehensive information regarding subjective wellbeing (Joo and Grable, 2004). Thus, FSE may be indirectly associated with life satisfaction through investment satisfaction. However, to the best of our knowledge, no studies have yet tested the mediating relationships between these variables in the finance domain.

### The Mediating Role of High Standards Tendency

Another potential mechanism underlying the association between FSE and life satisfaction is high standards tendency. High standards tendency is a psychological construct derived from the work of Schwartz et al. (2002), representing individuals’ tendencies to hold high standards for themselves and things in general. Relatively little research seems to have addressed this mediating process; however, some indirect evidence is available. First, FSE may increase one’s propensity for high standards. Given the motivational aspect of FSE, individuals high in FSE tend to believe in their capability to achieve financial goals. This belief might encourage them to maintain and enhance high standards and make further progress toward their financial goals, which are essential to high standards tendency (Schwartz et al., 2002; Lown, 2011). Moreover, some empirical studies have suggested a positive relationship between self-efficacy and high standards tendency (Ganske and Ashby, 2007; Lai, 2010; Rim et al., 2011). For example, Rim et al. (2011) reported that general self-efficacy was positively correlated with high standards tendency. Ganske and Ashby (2007) found that individuals with high self-efficacy for career decision-making were more likely to pursue high standards. These findings suggest that not only general self-efficacy but also domain-specific self-efficacy contribute to high standards tendency.

Additionally, researchers have argued that high standards tendency may positively influence individual life satisfaction. For example, Rim et al. (2011) and Purvis et al. (2011) found that high standards tendency was positively correlated with general life satisfaction. Moreover, high standards tendency is the core characteristic of adaptive perfectionism, which showed a positive association with general life satisfaction (Gilman et al., 2005; Park and Jeong, 2015). Therefore, FSE may be indirectly associated with life satisfaction through high standards tendency.

### The Relationship Between High Standards Tendency and Investment Satisfaction

As discussed above, both investment satisfaction and high standards tendency are associated with general life satisfaction. Additionally, in a study by Giacopelli et al. (2013), high standards tendency was found to significantly predict increased job satisfaction, suggesting a positive association between high standards tendency and domain-specific satisfaction. This finding also suggested that it is feasible to treat high standards tendency as a factor for domain-specific satisfaction. Nevertheless, there are few published studies to date on this topic. Additional research is therefore necessary to better understand the relationship between high standards tendency and satisfaction in other important domains. Thus, the present study aimed to address this gap in the literature by testing the hypothesis that high standards tendency would be significantly related to satisfaction in the investment domain.

### The Present Study

The present study sought to reveal the underlying mechanisms of the relationship between financial self-efficacy and general life satisfaction using a sequential mediation model with four specific goals: (1) to examine whether FSE, as domain-specific self-efficacy, is as significant predictor of general life satisfaction; (2) to examine whether investment satisfaction mediates the relationship between FSE and general life satisfaction; (3) to examine whether high standards tendency mediates the relationship between FSE and general life satisfaction, and (4) to determine whether high standards tendency is a significant independent predictor of investment satisfaction. Therefore, four hypotheses could be proposed as follows:

Hypothesis 1: FSE would relate positively to general life satisfaction.

Hypothesis 2: FSE would increase investment satisfaction, which in turn would contribute to general life satisfaction.

Hypothesis 3: FSE would improve high standards tendency, which in turn would contribute to general life satisfaction.

Hypothesis 4: FSE would sequentially increase high standards tendency and investment satisfaction, which in turn would contribute to general life satisfaction.

**Figure 1:**
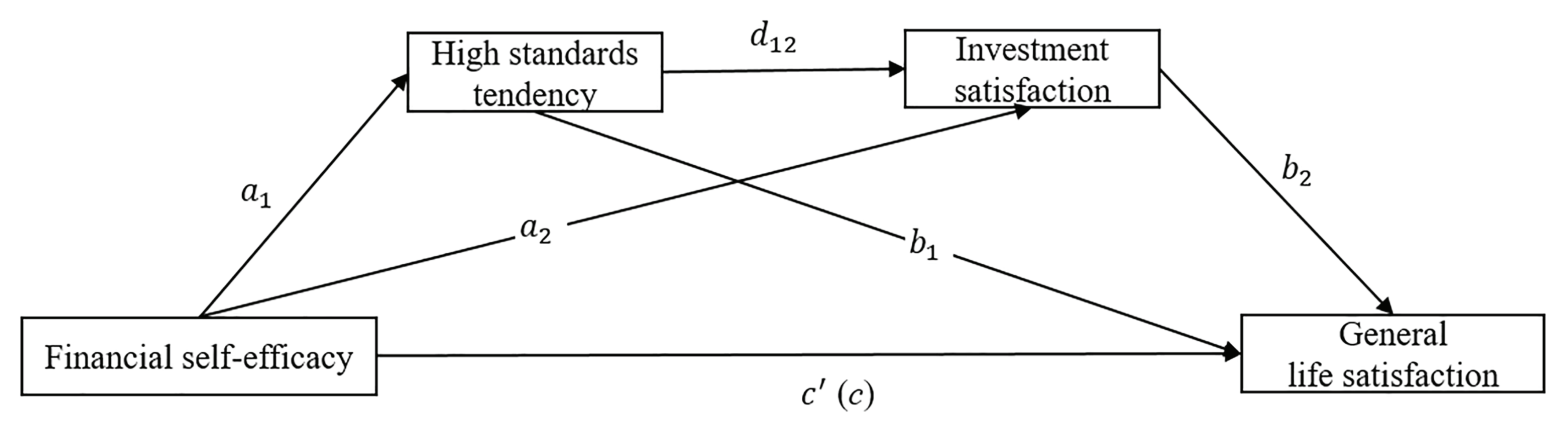
The hypothesized research model.

The hypothesized research model is presented in Figure 1.

## Materials and Methods

### Sample

Study participants included employees from the finance industry (e.g., banking, insurance). They were asked to complete questionnaires during their coffee break in training seminars and return them to the seminar instructors. The sample size was estimated using the G-power 3.1 program. For linear multiple regression, the minimum required number of participants was 279, based on an α level of 0.05, power (1-β) of 0.80, effect size (f^2^) of 0.05, and six predicting variables (Faul et al., 2009; Tang et al., 2019). Considering potential dropouts and missing data, questionnaires were distributed to 360 participants, and 342 were returned. Participants’ missing data on education (4.6%), age (0.9%), or gender (0.5%) were excluded from analysis. Thus, the final sample included 323 participants (172 males; mean age = 29.90 years, *SD* = 5.74). Written informed consent was obtained from all participants. All materials and procedures were approved by the Guangdong University of Finance Human Investigation Committee.

### Measures

#### Financial Self-Efficacy

Montford and Goldsmith (2016) developed the 5-item Financial Self-efficacy Scale. A Chinese version of the measurement was developed using forward and backward translation. It has demonstrated good reliability and validity (Tang et al., 2019). Participants indicated their degree of agreement to items using a 7-point scale ranging from “1” (strongly disagree) to “7” (strongly agree). The mean scores were computed, with higher scores indicating higher FSE. Cronbach’s alpha and McDonald’s Omega were 0.841 and 0.849, respectively, and the one dimensional structure was supported (CFI = 0.924, GFI = 0.909, SRMR = 0.050), demonstrating a good reliability and structure validity. The maximum shared squared variance (MSV, 0.893) was less than the average variance extracted (AVE, 0.901), and the AVE was no less than 0.50, indicating acceptable convergent validity and discriminant validity.

#### Global Life Satisfaction

Diener et al. (1985) developed the 5-item Life Satisfaction Scale. This scale has demonstrated good reliability and validity in Chinese samples (Du et al., 2015; Lian et al., 2018). Participants rated their degree of agreement to items using a 7-point scale ranging from “1” (strongly disagree) to “7” (strongly agree). The mean scores were computed, with higher scores indicating higher life satisfaction. Cronbach’s alpha and McDonald’s Omega were 0.931 and 0.931, respectively, and the one dimensional structure was supported (CFI = 0.983, GFI = 0.966, SRMR = 0.025), demonstrating a good reliability and structure validity. The MSV (0.900) was less than the AVE (0.950), and the AVE was no less than 0.50, indicating acceptable convergent validity and discriminant validity.

#### High Standards Tendency

High standards tendency was measured directly through one statement adopted from Pan and Statman (2012) and Nenkov et al. (2008). Participants were required to use a 7-point scale ranging from “1” (strongly disagree) to “7” (strongly agree) to rate how much they agreed with the following item: “No matter what I do, I have the highest standards for myself. Second best is not good enough for me.” Higher scores indicate more high standards tendency.

#### Investment Satisfaction

Investment satisfaction was measured directly through one statement adopted from Asif (2016) and Wang et al. (2006). Participants were required to use a 7-point scale ranging from “1” (strongly disagree) to “7” (strongly agree) to rate how much they agreed with the following item: “I was satisfied with trading results in the last year.” Higher scores indicate higher levels of investment satisfaction. Single-item measures of domain-specific satisfaction are appropriate for numerous research contexts and have been shown to be reliable and valid (Cho and Tay, 2016).

### Data Analysis

Data were analyzed using IBM SPSS version 22. Analyses were conducted in two steps. First, descriptive statistics (i.e., *M*, *SD*) and bivariate correlations for major variables were calculated. Second, to test the sequential mediation model, a bootstrapping method with an SPSS PROCESS Macro (Model 6; Hayes, 2017) was used. The 95% confidence interval (CI) for the indirect effect was a bias-corrected estimate based on 5,000 bootstrapping resamples. The mediating effect was considered to be significant at the level of *p* < 0.05, when the 95% CI did not include zero. Missing data were less than 1% and were estimated using expectation maximization.

## Results

### Preliminary Analyses

Correlation matrices for all variables along means and standard deviations are presented in Table 1. FSE, high standards tendency, and investment satisfaction showed significant and positive correlations with general life satisfaction. FSE and high standards tendency was positively associated with investment satisfaction. High standards tendency was positively related to general life satisfaction.

**Table 1:** Means and standard deviations of all variables along with their correlations.

Variables	M	SD	1	2	3	4	5	6	7
1. Gender	0.468	0.500	— —						
2. Age	29.898	5.744	−0.033	— —					
3. Education	4.102	0.615	−0.014	−0.005	— —				
4. Financial self-efficacy	4.622	1.053	−0.049	0.005	−0.084	— —			
5. High standards tendency	4.232	1.609	−0.089	−0.152^**^	−0.194^**^	0.605^**^	— —		
6. Investment satisfaction	4.015	1.675	0.036	0.033	−0.086	0.622^**^	0.489^**^	— —	
7. General life satisfaction	4.101	1.440	0.152^**^	0.071	−0.022	0.604^**^	0.449^**^	0.703^**^	— —

*N* = 323. Gender was dummy coded such that 0 = male and 1 = female. Education was coded as 1 = less than primary school and primary school, 2 = high school, 3 = college graduates, 4 = undergraduates, and 5 = master and above.

** *p* < 0.01.

### Mediation Analyses

**Figure 2:**
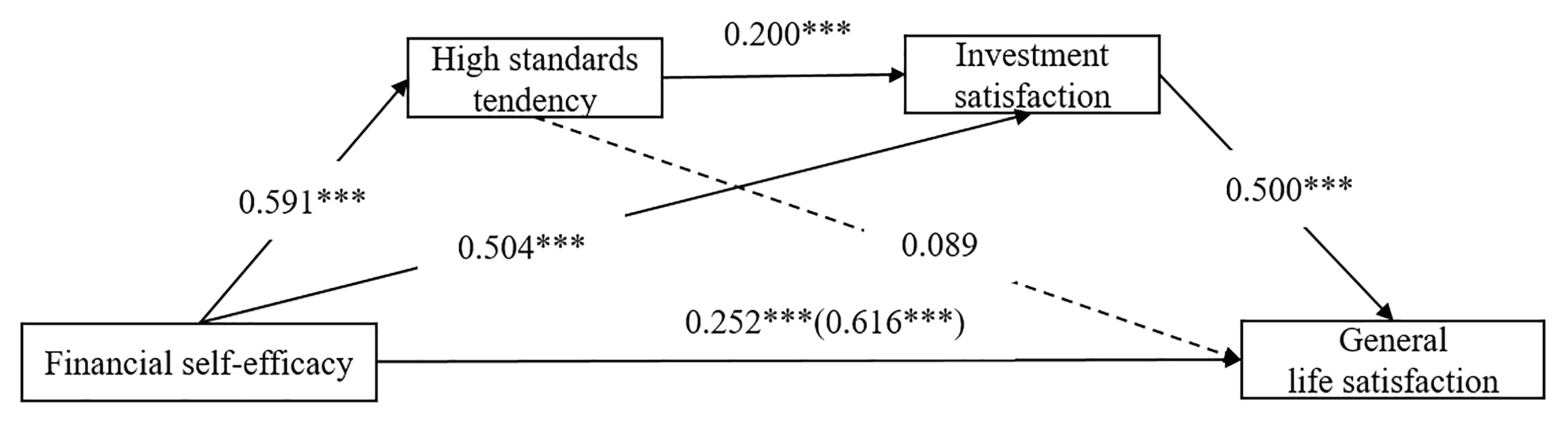
Effect of financial self-efficacy and general life satisfaction *via* high standards tendency and investment satisfaction. ^***^p < 0.001.

The Hayes SPSS Process Macro was used to examine the sequential mediating effect of high standards tendency and investment satisfaction in the relationship between FSE and general life satisfaction. The full process model showing all path coefficients is presented in Table 2 and Figure 2. Total, direct, and indirect effects are presented in Table 3.

**Table 2:** Testing the mediation effect of financial self-efficacy on general life satisfaction.

Predictors	Criterion: General life satisfaction			Criterion: High standards tendency			Criterion: Investment satisfaction			Criterion: General life satisfaction		
	*b*	*SE*	*t*	*b*	*SE*	*t*	*b*	*SE*	*t*	*b*	*SE*	*t*
Gender	0.372	0.087	4.283^***^	−0.135	0.086	−1.566	0.161	0.086	1.866	0.316	0.075	4.245^***^	
Age	0.075	0.043	1.724	−0.158	0.043	−3.690^***^	0.063	0.044	1.444	0.073	0.038	1.931	
Education	0.033	0.043	0.752	−0.146	0.043	−3.385^***^	−0.003	0.044	−0.078	0.062	0.038	1.645	
Financial self-efficacy	0.616	0.043	14.162^***^	0.591	0.043	13.710^***^	0.504	0.054	9.266^***^	0.252	0.053	4.794^***^	
High standards tendency							0.200	0.056	3.554^***^	0.089	0.049	1.815	
Investment satisfaction										0.500	0.048	10.367^***^	
R^2^	0.397			0.408			0.407		0.563			

*N* = 323.

*** *p* < 0.001.

**Table 3:** Total, direct, and indirect effects of financial self-efficacy on general life satisfaction.

	Effect	Standard error	95% CI	
			Lower	Upper
**Total effect**				
FSE→General life satisfaction	0.616	0.043		
**Direct effect**				
FSE→General life satisfaction	0.252	0.053	0.149	0.356
**Indirect effect**				
FSE→High standards tendency→General life satisfaction	0.053	0.036	−0.017	0.124
FSE→Investment satisfaction→General life satisfaction	0.252	0.042	0.177	0.339
FSE→High standards tendency→Investment satisfaction→General life satisfaction	0.059	0.020	0.025	0.107

CI, confidence interval.

First, as shown in Table 2, after controlling for the influence of gender, age, and education, FSE had a significant positive effect on general life satisfaction (*c* = 0.616, *p* < 0.001). Thus, Hypothesis 1 was supported. However, the effect of FSE on general life satisfaction was found to decrease (*c*’ = 0.252, *p* < 0.001) after the inclusion of high standards tendency and investment satisfaction in the direct effect model. This decrease in the magnitude of the effect of FSE on general life satisfaction without a decrease in the significance level indicated partial mediation.

Second, results of the indirect effect are displayed in Tables 2 and 3. After controlling for the influence of gender, age, and education, the effect of FSE on general life satisfaction through investment satisfaction was significant [a_2_b_2_ = 0.252, CI = (0.177, 0.339)]. Hypothesis 3 was supported. However, the indirect effect through high standards tendency was insignificant [a_1_b_1_ = 0.053, CI = (−0.017, 0.124)]. Hypothesis 2 was not supported. Further, the indirect effect of FSE on general life satisfaction through the sequential mediating effect of high standards tendency and investment satisfaction was significant [a_1_d_21_b_2_ = 0.059, CI = (0.025, 0.107)]. Hypothesis 4 was supported.

## Discussion

The present study aimed to investigate the influence of FSE on general life satisfaction. An attempt was also made to examine underlying psychological processes by testing a sequential mediation model. The results largely provided support for the study’s hypotheses. Individual FSE was found to relate positively with high standards tendency, investment satisfaction, and general life satisfaction. Additionally, FSE was found to influence general life satisfaction through investment satisfaction, and sequentially through high standards tendency and investment satisfaction. However, high standards tendency alone failed to mediate the relationship between FSE and general life satisfaction.

First, we found a positive relationship between FSE and general life satisfaction. Significant associations between domain-specific self-efficacy and general life satisfaction can be seen in different domains. For example, academic, social, health, and occupational self-efficacy have been shown to improve general life satisfaction (Vecchio et al., 2007). The findings of the present study suggested that the positive associations between domain-specific self-efficacy and general life satisfaction extend to the finance domain. FSE, which reflects the perceived control investors hold over themselves and their financial circumstances, enables investors to develop a constructive outlook on life.

Second, investment satisfaction was found to mediate the relationship between FSE and general life satisfaction. This mediation model provided compelling evidence for an integrative model of life satisfaction, which combines top-down and bottom-up explanations of life satisfaction (Heller et al., 2004). The first stage of mediation analysis revealed that FSE was associated with higher levels of investment satisfaction. The top-down approach treats domain-specific satisfaction and general life satisfaction as a function of the person, emphasizing the direct association between dispositional traits and domain/life satisfaction (Erdogan et al., 2012). Domain-specific self-efficacy can be considered a stable personality trait (Grether et al., 2018). The direct positive association between FSE and investment satisfaction indicated that FSE plays a highly prominent role in determining one’s satisfaction in the investment domain. At the second stage of mediation analysis, a positive association between investment satisfaction and general life satisfaction was found, congruent with the bottom-up approach, which posits that domain satisfaction information is used to make judgments regarding general life satisfaction. Satisfaction in different important life domains (e.g., social, health, occupational) has been found to directly influence general life satisfaction (Hart, 1999; Cho and Tay, 2016; Jovanović et al., 2017). Thus, the results of the present study extended findings from previous studies and suggested that satisfaction in the investment domain contributes to the explanation of general life satisfaction.

Third, high standards tendency failed to mediate the relationship between FSE and general life satisfaction; however, high standards tendency and investment satisfaction sequentially mediated this relationship. These findings may reveal the differential impact of high standards tendency on domain-specific life satisfaction and general life satisfaction. There is a possibility that the positive impact of high standards tendency may be more evident for specific tasks or goals than general ones, thus first leading to more satisfaction in a specific domain (e.g., the investment domain), which in turn contributes to more general life satisfaction. Another possible reason for these findings could be the use of a single-item measure for high standards tendency. A study to develop a scale related to high standards tendency also highlighted measurement issues in accurately capturing the construct (Nenkov et al., 2008). Additionally, the sequential mediation model provided new insights into the literature by revealing the possibilities of other pathways in explaining the relationship between domain-specific self-efficacy and general life satisfaction.

The present study has important practical implications. Searching for ways to improve general life satisfaction is an important personal and societal goal for both researchers and the general public (Diener, 2000). First, our model recognized the importance of FSE, regardless of participants’ sociodemographic background. Interventions should be tailored to help individuals enhance FSE. Since FSE is based on a person’s financial knowledge and skills, interventions targeting individuals with lower levels of FSE should be aimed at promoting financial literacy and improving financial decision-making skills (Williams, 2007; Rothwell et al., 2016). However, a debilitating effect of FSE may arise due to a lack of congruence between investors’ perceived capability to cope with financial circumstances and their levels of financial knowledge (Moores and Chang, 2009; Tang et al., 2019). These intervention programs may require a more accurate match between perceived FSE and actual financial literacy. Second, previous studies have identified the importance of domain-specific life satisfaction, such as work, social, and leisure, relatively neglecting the contributions of life satisfaction in finance, especially in the investment domain, although financial activities have become increasingly more relevant in consumers’ lives. With knowledge regarding high standards tendency and investment satisfaction as mediators operating sequentially in the link between FSE and general life satisfaction, targeting efforts toward proximal antecedents of general life satisfaction might be effective for improving general life satisfaction, particularly for situations where directly targeting FSE is more difficult. For example, practices to set high standards for investment decisions and performance in an adaptive way (Tashjian, 2019) and improve investment satisfaction (e.g., recognize and overcome the psychological biases in the financial investment decision making; Sahi, 2017) would be recommended to improve general life satisfaction.

We also acknowledge several limitations in the present study and suggest future research directions. First, we acknowledge that no conclusion regarding causality or directionality can be drawn, due to the study’s cross-sectional design. Reciprocal relationships between domain-specific satisfaction and general life satisfaction have been proposed (Lance et al., 1989; Singley et al., 2010); therefore, a longitudinal design may provide more comprehensive insight into the directionality among FSE, high standards tendency, investment satisfaction, and general life satisfaction. Second, self-reported data remain subject to several potential risks of bias, such as social desirability, selective memory bias, and common method variance. This limitation could be addressed by using multi-method and multi-informant data collection strategies. Third, this study used convenience sampling, which limits the ability to generalize beyond highly similar groups. Replication with a larger and more diverse sample should be done to validate the findings. Fourth, the present study only investigated the contributions of investment satisfaction to general life satisfaction, without controlling for the influence of satisfaction with other important domains, which may exaggerate the association between investment satisfaction and life satisfaction. Future research may benefit from simultaneously exploring contributions of different important satisfaction domains. Fifth, the present study used single items to assess high standards tendency and investment satisfaction. Multi-item measures have been shown to be preferable due to their superior psychometric properties. However, it is also worth noting that, as mentioned in the Methods, single-item measures of domain-specific satisfaction are appropriate for numerous research contexts and have been shown to be reliable and valid. For a relatively intuitive construct such as high standards tendency, single-item indices can be as informative as multi-item scales, although single-item measures of high standards tendency have been used in relatively few studies (Burisch, 1984; Nenkov et al., 2008; Pan and Statman, 2012). The development and use of brief and multi-item high standards tendency measures is encouraged for future research.

Despite the above limitations, the present study provided empirical evidence for a model in which high standards tendency and investment satisfaction sequentially mediated the relationship between FSE and general life satisfaction. Thus, contributions to positive psychology can be drawn from novel insights from our work to help individuals improve general and domain-specific life satisfaction.

## Data Availability Statement

The raw data supporting the conclusions of this article will be made available by the authors, without undue reservation.

## Ethics Statement

The studies involving human participants were reviewed and approved by Guangdong University of Finance Human Investigation Committee. The patients/participants provided their written informed consent to participate in this study.

## Author Contributions

JH and ST conceived the research. JH, MD, and ST designed the research. LQ, YW, and JH performed the research and analyzed the data. JH, LQ, YW, JZ, MD, ST, and WZ contributed to the writing of the manuscript. All authors contributed to the article and approved the submitted version.

### Conflict of Interest

The authors declare that the research was conducted in the absence of any commercial or financial relationships that could be construed as a potential conflict of interest.

## References

[ref1] Asif M. (2016). Behavioral biases and their impact on the satisfaction of the investor: a case of small investors of Lahore stock exchange. Middle East J. Bus. 11, 3–11. 10.5742/MEJB.2016.92832

[ref2] Azizli N. Atkinson B. E. Baughman H. M. Giammarco E. A. (2015). Relationships between general self-efficacy, planning for the future, and life satisfaction. Personal. Individ. Differ. 82, 58–60. 10.1016/j.paid.2015.03.006

[ref3] Badri M. A. Mohaidat J. Ferrandino V. El Mourad T. (2013). The social cognitive model of job satisfaction among teachers: testing and validation. Int. J. Educ. Res. 57, 12–24. 10.1016/j.ijer.2012.10.007

[ref4] Bandura A. (1989). Human agency in social cognitive theory. Am. Psychol. 44, 1175–1184. 10.1037/0003-066X.44.9.1175, PMID: 2782727

[ref5] Bandura A. (1997). Self-efficacy: The exercise of control. New York, NY: Freeman.

[ref6] Brown S. P. Jones E. Leigh T. W. (2005). The attenuating effect of role overload on relationships linking self-efficacy and goal level to work performance. J. Appl. Psychol. 90, 972–979. 10.1037/0021-9010.90.5.972, PMID: 16162069

[ref7] Burger K. Samuel R. (2017). The role of perceived stress and self-efficacy in young people’s life satisfaction: a longitudinal study. J. Youth Adolesc. 46, 78–90. 10.1007/s10964-016-0608-x, PMID: 27812840

[ref8] Burisch M. (1984). Approaches to personality inventory construction: a comparison of merits. Am. Psychol. 39:214. 10.1037/0003-066X.39.3.214

[ref9] Cho E. Tay L. (2016). Domain satisfaction as a mediator of the relationship between work–family spillover and subjective well-being: a longitudinal study. J. Bus. Psychol. 31, 445–457. 10.1007/s10869-015-9423-8

[ref10] Diener E. (2000). Subjective well-being: the science of happiness and a proposal for a national index. Am. Psychol. 55, 34–43. 10.1037/0003-066X.55.1.34, PMID: 11392863

[ref11] Diener E. Emmons R. A. Larsen R. J. Griffin S. (1985). The satisfaction with life scale. J. Pers. Assess. 49, 71–75. 10.1207/s15327752jpa4901_13 16367493

[ref12] Donnelly G. Iyer R. Howell R. T. (2012). The big five personality traits, material values, and financial well-being of self-described money managers. J. Econ. Psychol. 33, 1129–1142. 10.1016/j.joep.2012.08.001

[ref13] Du H. Bernardo A. B. Yeung S. S. (2015). Locus-of-hope and life satisfaction: the mediating roles of personal self-esteem and relational self-esteem. Personal. Individ. Differ. 83, 228–233. 10.1016/j.paid.2015.04.026

[ref14] Erdogan B. Bauer T. N. Truxillo D. M. Mansfield L. R. (2012). Whistle while you work: a review of the life satisfaction literature. J. Manag. 38, 1038–1083. 10.1177/0149206311429379

[ref15] Farrell L. Fry T. R. Risse L. (2016). The significance of financial self-efficacy in explaining women’s personal finance behaviour. J. Econ. Psychol. 54, 85–99. 10.1016/j.joep.2015.07.001

[ref16] Faul F. Erdfelder E. Buchner A. Lang A. -G. (2009). Statistical power analyses using G* Power 3.1: tests for correlation and regression analyses. Behav. Res. Methods 41, 1149–1160. 10.3758/BRM.41.4.1149, PMID: 19897823

[ref17] Fergusson D. McLeod G. Horwood L. J. Swain N. Chapple S. Poulton R. (2015). Life satisfaction and mental health problems (18 to 35 years). Psychol. Med. 45, 2427–2436. 10.1017/S0033291715000422, PMID: 25804325

[ref18] Forbes J. Kara S. M. (2010). Confidence mediates how investment knowledge influences investing self-efficacy. J. Econ. Psychol. 31, 435–443. 10.1016/j.joep.2010.01.012

[ref19] Ganske K. H. Ashby J. S. (2007). Perfectionism and career decision-making self-efficacy. J. Employ. Couns. 44, 17–28. 10.1002/j.2161-1920.2007.tb00021.x

[ref20] Garriott P. O. Hudyma A. Keene C. Santiago D. (2015). Social cognitive predictors of first-and non-first-generation college students’ academic and life satisfaction. J. Couns. Psychol. 62:253. 10.1037/cou0000066, PMID: 25730170

[ref21] Giacopelli N. M. Simpson K. M. Dalal R. S. Randolph K. L. Holland S. J. (2013). Maximizing as a predictor of job satisfaction and performance: a tale of three scales. Judgm. Decis. Mak. 8, 448–469.

[ref22] Gigantesco A. Fagnani C. Toccaceli V. Stazi M. A. Lucidi F. Violani C.. (2019). The relationship between satisfaction with life and depression symptoms by gender. Front. Psych. 10:419. 10.3389/fpsyt.2019.00419, PMID: 31258495 PMC6588028

[ref23] Gilman R. Ashby J. S. Sverko D. Florell D. Varjas K. (2005). The relationship between perfectionism and multidimensional life satisfaction among Croatian and American youth. Personal. Individ. Differ. 39, 155–166. 10.1016/j.paid.2004.12.014

[ref24] Ginevra M. C. Magnano P. Lodi E. Annovazzi C. Camussi E. Patrizi P.. (2018). The role of career adaptability and courage on life satisfaction in adolescence. J. Adolesc. 62, 1–8. 10.1016/j.adolescence.2017.11.002, PMID: 29127913

[ref25] Grether T. Sowislo J. F. Wiese B. S. (2018). Top-down or bottom-up? Prospective relations between general and domain-specific self-efficacy beliefs during a work-family transition. Personal. Individ. Differ. 121, 131–139. 10.1016/j.paid.2017.09.021

[ref26] Gutter M. Copur Z. (2011). Financial behaviors and financial well-being of college students: evidence from a national survey. J. Fam. Econ. Iss. 32, 699–714. 10.1007/s10834-011-9255-2

[ref27] Hart P. M. (1999). Predicting employee life satisfaction: a coherent model of personality, work, and nonwork experiences, and domain satisfactions. J. Appl. Psychol. 84:564. 10.1037/0021-9010.84.4.564

[ref28] Hayes A. F. (2017). Introduction to mediation, moderation, and conditional process analysis: A regression-based approach. New York, NY: Guilford Publications.

[ref29] Heffner A. L. Antaramian S. P. (2016). The role of life satisfaction in predicting student engagement and achievement. J. Happiness Stud. 17, 1681–1701. 10.1007/s10902-015-9665-1

[ref69] Heller D. Watson D. Ilies R. (2004). The role of person versus situation in life satisfaction: a critical examination. Psychol. Bull. 130:574. 10.1037/0033-2909.130.4.574 15250814

[ref30] Joo S. -H. Grable J. E. (2004). An exploratory framework of the determinants of financial satisfaction. J. Fam. Econ. Iss. 25, 25–50. 10.1023/B:JEEI.0000016722.37994.9f

[ref31] Jovanović V. Joshanloo M. Đunda D. Bakhshi A. (2017). Gender differences in the relationship between domain-specific and general life satisfaction: a study in Iran and Serbia. Appl. Res. Qual. Life 12, 185–204. 10.1007/s11482-016-9461-z

[ref32] Korniotis G. M. Kumar A. (2011). Do older investors make better investment decisions? Rev. Econ. Stat. 93, 244–265. 10.1162/REST_a_00053

[ref33] Lai L. (2010). Maximizing without difficulty: a modified maximizing scale and its correlates. Judgm. Decis. Mak. 5:164.

[ref34] Lance C. E. Lautenschlager G. J. Sloan C. E. Varca P. E. (1989). A comparison between bottom–up, top–down, and bidirectional models of relationships between global and life facet satisfaction. J. Pers. 57, 601–624. 10.1111/j.1467-6494.1989.tb00565.x

[ref35] Lee J. C. Mortimer J. T. (2009). Family socialization, economic self-efficacy, and the attainment of financial independence in early adulthood. Longit. Life Course Stud. 1:45. PMID: 22025928 PMC3198812

[ref36] Lent R. W. (2004). Toward a unifying theoretical and practical perspective on well-being and psychosocial adjustment. J. Couns. Psychol. 51, 482–509. 10.1037/0022-0167.51.4.482

[ref37] Lent R. W. Singley D. Sheu H. -B. Gainor K. A. Brenner B. R. Treistman D.. (2005). Social cognitive predictors of domain and life satisfaction: exploring the theoretical precursors of subjective well-being. J. Couns. Psychol. 52, 429–442. 10.1037/0022-0167.52.3.429

[ref38] Lian S. -L. Sun X. -J. Yang X. -j. Zhou Z. -K. (2018). The effect of adolescents’ active social networking site use on life satisfaction: the sequential mediating roles of positive feedback and relational certainty. Curr. Psychol. 39, 1–9. 10.1007/s12144-018-9882-y

[ref39] Lown J. M. (2011). Development and validation of a financial self-efficacy scale. *J. Financial Couns. Plan.* 22:54.

[ref40] Montford W. Goldsmith R. E. (2016). How gender and financial self-efficacy influence investment risk taking. Int. J. Consum. Stud. 40, 101–106. 10.1111/ijcs.12219

[ref41] Moores T. T. Chang J. C. -J. (2009). Self-efficacy, overconfidence, and the negative effect on subsequent performance: a field study. Inf. Manag. 46, 69–76. 10.1016/j.im.2008.11.006

[ref42] Nenkov G. Y. Morrin M. Schwartz B. Ward A. Hulland J. (2008). A short form of the maximization scale: factor structure, reliability and validity studies. Judgm. Decis. Mak. 3, 371–388. 10.1007/s10902-007-9055-4

[ref43] Ng W. Diener E. (2014). What matters to the rich and the poor? Subjective well-being, financial satisfaction, and postmaterialist needs across the world. J. Pers. Soc. Psychol. 107:326. 10.1037/a0036856, PMID: 25090131

[ref44] Paciello M. Ghezzi V. Tramontano C. Barbaranelli C. Fida R. (2016). Self-efficacy configurations and wellbeing in the academic context: a person-centred approach. Personal. Individ. Differ. 99, 16–21. 10.1016/j.paid.2016.04.083

[ref45] Pan C. H. Statman M. (2012). Questionnaires of risk tolerance, regret, overconfidence, and other investor propensities. J. Invest. Consul. 13, 54–63. 10.2139/ssrn.1549912

[ref46] Park H. -j. Jeong D. Y. (2015). Psychological well-being, life satisfaction, and self-esteem among adaptive perfectionists, maladaptive perfectionists, and nonperfectionists. Personal. Individ. Differ. 72, 165–170. 10.1016/j.paid.2014.08.031

[ref47] Paschali A. Tsitsas G. (2010). *Stress and life satisfaction among university students-a pilot study*. Paper presented at the Annals of general psychiatry.

[ref48] Pavot W. Diener E. (1993). The affective and cognitive context of self-reported measures of subjective well-being. Soc. Indic. Res. 28, 1–20. 10.1007/BF01086714

[ref49] Peterson C. Park N. Seligman M. E. (2005). Orientations to happiness and life satisfaction: the full life versus the empty life. J. Happiness Stud. 6, 25–41. 10.1007/s10902-004-1278-z

[ref50] Purvis A. Howell R. T. Iyer R. (2011). Exploring the role of personality in the relationship between maximization and well-being. Personal. Individ. Differ. 50, 370–375. 10.1016/j.paid.2010.10.023

[ref51] Rim H. B. Turner B. M. Betz N. E. Nygren T. E. (2011). Studies of the dimensionality, correlates, and meaning of measures of the maximizing tendency. Judgm. Decis. Mak. 6:565. 10.1007/s10936-011-9171-5

[ref52] Rothwell D. W. Khan M. N. Cherney K. (2016). Building financial knowledge is not enough: financial self-efficacy as a mediator in the financial capability of low-income families. J. Community Pract. 24, 368–388. 10.1080/10705422.2016.1233162

[ref53] Russo-Netzer P. Horenczyk G. Bergman Y. S. (2019). Affect, meaning in life, and life satisfaction among immigrants and non-immigrants: a moderated mediation model. Curr. Psychol. 1–9. 10.1007/s12144-019-00284-z

[ref54] Sahi S. K. (2017). Psychological biases of individual investors and financial satisfaction. J. Consum. Behav. 16, 511–535. 10.1002/cb.1644

[ref55] Schwartz B. Ward A. Monterosso J. Lyubomirsky S. White K. Lehman D. R. (2002). Maximizing versus satisficing: happiness is a matter of choice. J. Pers. Soc. Psychol. 83:1178. 10.1037/0022-3514.83.5.1178, PMID: 12416921

[ref56] Sheu H. -B. Mejia A. Rigali-Oiler M. Primé D. R. Chong S. S. (2016). Social cognitive predictors of academic and life satisfaction: measurement and structural equivalence across three racial/ethnic groups. J. Couns. Psychol. 63:460. 10.1037/cou0000158, PMID: 27177025

[ref57] Singley D. B. Lent R. W. Sheu H. -B. (2010). Longitudinal test of a social cognitive model of academic and life satisfaction. J. Career Assess. 18, 133–146. 10.1177/1069072709354199

[ref58] Steger M. F. Oishi S. Kesebir S. (2011). Is a life without meaning satisfying? The moderating role of the search for meaning in satisfaction with life judgments. J. Posit. Psychol. 6, 173–180. 10.1080/17439760.2011.569171

[ref59] Suldo S. M. Huebner E. S. (2006). Is extremely high life satisfaction during adolescence advantageous? Soc. Indic. Res. 78, 179–203. 10.1007/s11205-005-8208-2

[ref60] Sun R. C. Shek D. T. (2010). Life satisfaction, positive youth development, and problem behaviour among Chinese adolescents in Hong Kong. Soc. Indic. Res. 95, 455–474. 10.1007/s11205-009-9531-9, PMID: 20062815 PMC2801834

[ref61] Tang S. Huang S. Zhu J. Huang R. Tang Z. Hu J. (2019). Financial self-efficacy and disposition effect in investors: the mediating role of versatile cognitive style. Front. Psychol. 9:2705. 10.3389/fpsyg.2018.02705, PMID: 30671010 PMC6331392

[ref62] Tashjian E. (2019). The essence of investing: experiential education with a student-run portfolio. Manag. Financ. 46, 530–547. 10.1108/MF-08-2018-0401

[ref63] Vecchio G. M. Gerbino M. Pastorelli C. Del Bove G. Caprara G. V. (2007). Multi-faceted self-efficacy beliefs as predictors of life satisfaction in late adolescence. Personal. Individ. Differ. 43, 1807–1818. 10.1016/j.paid.2007.05.018

[ref64] Wang X. L. Shi K. Fan H. X. (2006). Psychological mechanisms of investors in Chinese stock markets. J. Econ. Psychol. 27, 762–780. 10.1016/j.joep.2006.06.007

[ref65] Weber M. Huebner E. S. (2015). Early adolescents’ personality and life satisfaction: a closer look at global vs. domain-specific satisfaction. Personal. Individ. Differ. 83, 31–36. 10.1016/j.paid.2015.03.042

[ref66] Williams T. (2007). Empowerment of whom and for what? Financial literacy education and the new regulation of consumer financial services. Law Policy 29, 226–256. 10.1111/j.1467-9930.2007.00254.x

[ref67] Xiao J. J. Chatterjee S. Kim J. (2014a). Factors associated with financial independence of young adults. Int. J. Consum. Stud. 38, 394–403. 10.1111/ijcs.12106

[ref68] Xiao J. J. Chen C. Chen F. (2014b). Consumer financial capability and financial satisfaction. Soc. Indic. Res. 118, 415–432. 10.1007/s11205-013-0414-8

